# The Burden of Opioid-Related Mortality in the United States

**DOI:** 10.1001/jamanetworkopen.2018.0217

**Published:** 2018-06-01

**Authors:** Tara Gomes, Mina Tadrous, Muhammad M. Mamdani, J. Michael Paterson, David N. Juurlink

**Affiliations:** 1 Keenan Research Centre of the Li Ka Shing Knowledge Institute, St. Michael’s Hospital, Toronto, Ontario, Canada; 2 Institute for Clinical Evaluative Sciences, Toronto, Ontario, Canada; 3 Sunnybrook Research Institute, Toronto, Ontario, Canada

## Abstract

**Importance:**

Opioid prescribing and overdose are leading public health problems in North America, yet the precise public health burden has not been quantified.

**Objective:**

To examine the burden of opioid-related mortality across the United States over time.

**Design, Setting, and Participants:**

This study used a serial cross-sectional design in which cross sections were examined at different time points to investigate deaths from opioid-related causes in the United States between January 1, 2001, and December 31, 2016.

**Main Outcomes and Measures:**

Opioid-related deaths, defined as those in which a prescription or illicit opioid contributed substantially to an individual’s cause of death as determined by death certificates. We compared the percentage of deaths attributable to opioids and the associated person-years of life lost by age group.

**Results:**

Between 2001 and 2016, the number of opioid-related deaths in the United States increased by 345%, from 9489 to 42 245 deaths (33.3 to 130.7 deaths per million population). By 2016, men accounted for 67.5% of all opioid-related deaths, and the median (interquartile range) age at death was 40 (30-52) years. The percentage of deaths attributable to opioids increased in a similar fashion. In 2001, 0.4% of deaths (1 in 255) were opioid related, rising to 1.5% of deaths (1 in 65) by 2016, an increase of 292%. This burden was highest among adults aged 24 to 35 years. In this age group, 20.0% of deaths were attributable to opioids in 2016. Among those aged 15 to 24 years, 12.4% of deaths were attributable to opioids in 2016. Overall, opioid-related deaths resulted in 1 681 359 years of life lost (5.2 per 1000 population) in the United States in 2016, most of which (1 125 711 years of life lost) were among men. Adults aged 25 to 34 years had 12.9 years of life lost per 1000 population, and those aged 35 to 44 years had 9.9 years of life lost per 1000 population.

**Conclusions and Relevance:**

Premature death from opioid-related causes imposes an enormous public health burden across the United States. The recent increase in deaths attributable to opioids among those aged 15 to 34 years highlights a need for targeted programs and policies that focus on improved addiction care and harm reduction measures in this high-risk population.

## Key Points

**Question:**

What has been the burden of opioid-related deaths in the United States over a recent 15-year period?

**Findings:**

In this serial cross-sectional study, we found that the percentage of all deaths attributable to opioids increased 292% (from 0.4% to 1.5%) between 2001 and 2016, resulting in approximately 1.68 million person-years of life lost in 2016 alone (5.2 per 1000 population). The burden was particularly high among adults aged 24 to 35 years; in 2016, 20% of deaths in this age group involved opioids.

**Meaning:**

Premature death from opioids imposes an enormous and growing public health burden across the United States.

## Teaser

This population epidemiology study uses Centers for Disease Control and Prevention (CDC) mortality data to characterize trends in opioid-related deaths and estimate years of life lost due to opioids in the United States between 2001 and 2016.

## Introduction

Increased rates of opioid prescribing and the associated negative health consequences have emerged as leading public health problems in North America,^1^ particularly among young and middle-aged adults.^2^ By 2014, Canada and the United States had the highest per capita opioid consumption in the world,^3^ and deaths related to opioid use have increased dramatically in both countries.^2,4,5^ Importantly, opioid-related death rates are increasing most quickly among adults aged 25 to 44 years in the United States.^2^ Consequently, the public health burden resulting from early loss of life is substantial.

Despite this, there is little recent information on the burden of opioid-related deaths in the United States. One study measuring the burden of opioid addiction globally in 2010 found that in North America, opioid dependence was responsible for nearly 1 million disability-adjusted life-years annually.^6^ This study also found that more than half of disability-adjusted life-years were attributable to years of life lost (YLL),^6^ indicating the important role that early loss of life has when quantifying the burden of opioid-related harm. In the United States, prescription opioid overdose contributed to 830 652 YLL among people younger than 65 years in 2008^7^; however, this estimate was based on crude estimates of burden. Given the rapidly rising rate of opioid-related death across the United States, as well as the dramatically increasing role of fentanyl and other illicit opioids, more recent estimates of this burden are required. We therefore sought to quantify the burden of opioid-related death in the United States, and to compare this burden over time and by age and sex.

## Methods

### Study Setting and Population

This study used a serial cross-sectional design in which repeated cross sections were examined over time to determine the number of opioid-related deaths in the United States between 2001 and 2016. We used the Centers for Disease Control and Prevention (CDC) WONDER Multiple Cause of Death Online Database, which captures mortality and population estimates across the United States stratified by age and sex. This database captures cause of death data from death certificates of US residents and includes both the primary underlying cause of death and up to 20 additional causes of death.^8^ This study was approved by the research ethics board of Sunnybrook Health Sciences Centre, Toronto, Ontario, Canada, and follows the Strengthening the Reporting of Observational Studies in Epidemiology (STROBE) guidelines for reporting. Patient informed consent was not required due to the deidentified, aggregated nature of the data.

### Opioid-Related Deaths

We obtained population-based estimates of all deaths related to prescribed or illicit (eg, heroin and fentanyl derivatives) opioids in the United States using the CDC WONDER database. Consistent with the CDC,^9^ we defined opioid-related deaths as those with an underlying cause of death related to poisoning (*International Statistical Classification of Diseases and Related Health Problems, 10th Revision* [*ICD-10*] codes X40-X44, X60-64, X85, and Y10-Y14) and a multiple cause of death code related to an opioid (*ICD-10* codes T40.0-T40.4 and T40.6), excluding all deaths for which the age of the deceased person was unknown.

### Burden of Opioid-Related Deaths

To explore the burden of opioid-related deaths by age, we stratified patients into 7 age groups at the time of death: 0 to 14 years, 15 to 24 years, 25 to 34 years, 35 to 44 years, 45 to 54 years, 55 to 64 years, and 65 years or older. We quantified the burden of opioid-related death in 2 ways. First, we identified the proportion of all deaths in each age group that were opioid related using age-specific all-cause mortality estimates as the denominator. Second, we calculated the YLL due to opioid-related death using methods adapted from the Global Burden of Disease study.^10^ We calculated YLL using standard 5-year life expectancy tables developed by the World Health Organization and did not apply discounting or age weights to align with the organization’s most recent guidance.^10^ We calculated YLL for men and women by multiplying the number of deaths by the standard life expectancy and adjusting for the average age at death within each 5-year age group. The YLL were aggregated into 10-year age groups overall and stratified by sex.

### Statistical Analysis

We used descriptive statistics to summarize demographic characteristics of opioid-related deaths over the study period (numbers and percentages for binary variables, and median and interquartile range for continuous variables). In our analysis of the age-specific proportion of deaths attributable to opioids, we compared percentages in the years 2001, 2006, 2011, and 2016 using the Cochran-Armitage test for trend for each age group. All analyses used a 2-sided type I error rate of .05 as the threshold for statistical significance.

## Results

Over the 15-year study period, 335 123 opioid-related deaths in the United States met our inclusion criteria, with an increase of 345% from 9489 in 2001 (33.3 deaths per million population) to 42 245 in 2016 (130.7 deaths per million population). By 2016, men accounted for 67.5% of all opioid-related deaths (n = 28 496), and the median (interquartile range) age at death was 40 (30-52) years. The proportion of deaths attributable to opioids increased over the study period, rising 292% (from 0.4% [1 in 255] to 1.5% [1 in 65]), and increased steadily over time in each age group studied (*P* < .001 for all age groups) (Figure). The largest absolute increase between 2001 and 2016 was observed among those aged 25 to 34 years (15.8% increase from 4.2% in 2001 to 20.0% in 2016), followed by those aged 15 to 24 years (9.4% increase from 2.9% to 12.4%). However, the largest relative increases occurred among adults aged 55 to 64 years (754% increase from 0.2% to 1.7%) and those aged 65 years and older (635% increase from 0.01% to 0.07%). Despite the fact that confirmed opioid-related deaths represent a small percentage of all deaths in these older age groups, the absolute number of deaths is moderate. In 2016, 18.4% (7762 of 42 245) of all opioid-related deaths in the United States occurred among those aged 55 years and older.

**Figure.**
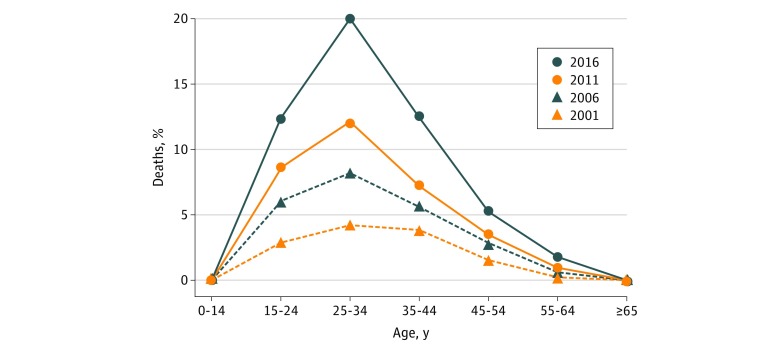
Proportion of Deaths Related to Opioid Use by Age Group in 2001, 2006, 2011, and 2016 The proportion of deaths in each age group that involved an opioid was calculated using opioid-related death data and all-cause mortality data. Death data were obtained from the Centers for Disease Control and Prevention WONDER online database. This analysis was performed at four 5-year time intervals between 2001 and 2016.

In our analysis of the burden of early loss of life from opioid overdose, we found that opioid-related deaths were responsible for 1 681 359 YLL (5.2 YLL per 1000 population) in the United States in 2016 (Table); however, this varied by age and sex. In particular, when stratified by age, adults aged 25 to 34 years and those aged 35 to 44 years experienced the highest burden from opioid-related deaths (12.9 YLL per 1000 population and 9.9 YLL per 1000 population, respectively). We also found that the burden of opioid-related death was higher among men (1 125 711 YLL; 7.0 YLL per 1000 population) compared with women (555 648 YLL; 3.4 YLL per 1000 population). Importantly, among men aged 25 to 34 years, this rate increased to 18.1 YLL per 1000 population, and the total YLL in this population represented nearly one-quarter of all YLL in the United States in 2016 (411 805 of 1 681 359 [24.5%]).

**Table.** Years of Life Lost (YLL) From Opioid-Related Deaths in the United States in 2016

Sex and Age	US Population, No.	Opioid-Related Deaths, No.	Rate of Opioid-Related Deaths, No./1 000 000 Population	YLL, No.	YLL, No./1000 Population
Men					
0-14 y	31 182 660	39	1.3	2851	0.1
15-24 y	22 360 342	2986	133.5	167 455	7.5
25-34 y	22 762 306	8436	370.6	411 805	18.1
35-44 y	20 249 194	6701	330.9	267 824	13.2
45-54 y	21 159 291	5658	267.4	173 710	8.2
55-64 y	20 081 837	3861	192.3	89 535	4.5
≥65 y	21 895 128	815	37.2	12 530	0.6
Total	159 690 758	28 496	178.4	1 125 711	7.0
Women					
0-14 y	29 854 687	44	1.5	3329	0.1
15-24 y	21 252 215	1041	49.0	63 240	3.0
25-34 y	22 102 199	3116	141.0	164 871	7.5
35-44 y	20 328 343	3046	149.8	132 787	6.5
45-54 y	21 705 077	3416	157.4	116 443	5.4
55-64 y	21 536 994	2460	114.2	64 529	3.0
≥65 y	27 525 255	626	22.7	10 449	0.4
Total	164 304 770	13 749	83.7	555 648	3.4
Overall					
0-14 y	61 037 347	83	1.4	6180	0.1
15-24 y	43 612 557	4027	92.3	230 694	5.3
25-34 y	44 864 505	11 552	257.5	576 676	12.9
35-44 y	40 577 537	9747	240.2	400 611	9.9
45-54 y	42 864 368	9074	211.7	290 153	6.8
55-64 y	41 618 831	6321	151.9	154 065	3.7
≥65 y	49 420 383	1441	29.2	22 979	0.5
Total	323 995 528	42 245	130.4	1 681 359	5.2

## Discussion

In this population-based study of the burden of opioid-related deaths in the United States, we found that 1 in 65 deaths was opioid related in 2016, representing an enormous toll in YLL. Indeed, in the United States, the YLL from opioid-related deaths exceed those attributable to hypertension, HIV/AIDS, and pneumonia and amount to approximately one-tenth of the YLL due to cancer.^11,12^ Furthermore, this burden is highest among adults aged 25 to 34 years; in this age group, 1 in 5 deaths in the United States is opioid related.

These findings highlight changes in the burden of opioid-related deaths over time and across demographic groups in the United States. They demonstrate the important role of opioid overdose in deaths of adolescents and young adults as well as the disproportionate burden of overdose among men. Furthermore, despite the fact that opioid-related deaths do not represent a large proportion of deaths among adults aged 55 years and older, the relative increase in recent years requires attention, as it could be indicative of an aging population with increasing prevalence of opioid use disorder. This is particularly problematic as recent estimates from the United States suggest that the prevalence of opioid misuse among adults aged 50 years and older is expected to double (from 1.2% to 2.4%) between 2004 and 2020.^13^

### Limitations

This study uses regularly collected population-based data from death certificates of US residents over a 15-year study period to generate population-based estimates of the current and changing burden of opioid-related deaths. One limitation that merits emphasis is the validity of our outcome definition. Death investigations are managed at the state level and generally consist of a medical examiner system, coroner system, or a combination of the two.^14^ These systems may lead to differential ascertainment of opioid-related deaths across the country because the determination of opioid involvement relies on the quality of the death investigation, including whether autopsy and postmortem toxicology results are obtained. Therefore, because the absence of postmortem toxicology results could inappropriately misclassify some deaths as not opioid related, it is likely that our results underestimate the true burden of opioid-related death across the country. Despite this, the definition used in this study is consistent with reporting of opioid-related deaths that is regularly conducted by the CDC,^9^ and these findings are therefore comparable with other regularly reported national statistics.

## Conclusions

Premature death from opioid-related causes imposes an enormous and growing public health burden across the United States. The recent increase in the proportion of deaths attributable to opioids among adolescents and young adults and the accompanying estimates of YLL are alarming, particularly among men. Furthermore, the aging population of people with opioid use disorder requires attention, as the burden of opioid overdose among adults aged 55 to 64 years is growing at a concerning rate. These trends highlight a need for tailored programs and policies that focus on both appropriate prescribing and harm reduction in these demographics.

## References

[1] Office of the Assistant Secretary for Planning and Evaluation Opioid abuse in the U.S. and HHS actions to address opioid-drug related overdoses and deaths. https://aspe.hhs.gov/basic-report/opioid-abuse-us-and-hhs-actions-address-opioid-drug-related-overdoses-and-deaths. Published March 26, 2015. Accessed May 4, 2017.

[2] Rudd RA, Seth P, David F, Scholl L Increases in drug and opioid-involved overdose deaths—United States, 2010-2015. MMWR Morb Mortal Wkly Rep. 2016;65(5051):-. 28033313 10.15585/mmwr.mm655051e1

[3] Pain and Policy Studies Group Opioid consumption motion chart. 2016 https://ppsg.medicine.wisc.edu/chart. Accessed November 27, 2016.

[4] Gomes T, Greaves S, Martins D, Latest Trends in Opioid-Related Deaths in Ontario: 1991 to 2015. Toronto, ON: Ontario Drug Policy Research Network; 2017.

[5] British Columbia Coroners Service Illicit Drug Overdose Deaths in BC. January 1, 2007-March 31, 2017. Burnaby, BC: British Columbia Coroners Service; 2017.

[6] Degenhardt L, Charlson F, Mathers B, The global epidemiology and burden of opioid dependence: results from the global burden of disease 2010 study. Addiction. 2014;109(8):1320-1333. 24661272 10.1111/add.12551

[7] Centers for Disease Control and Prevention (CDC) Vital signs: overdoses of prescription opioid pain relievers—United States, 1999-2008. MMWR Morb Mortal Wkly Rep. 2011;60(43):1487-1492. 22048730

[8] Centers for Disease Control and Prevention, National Center for Health Statistics Multiple cause of death files, 1999-2016. CDC WONDER Online Database. https://wonder.cdc.gov/mcd-icd10.html. Published December 2017. Accessed March 6, 2018.

[9] Rudd RA, Aleshire N, Zibbell JE, Gladden RM Increases in drug and opioid overdose deaths—United States, 2000-2014. MMWR Morb Mortal Wkly Rep. 2016;64(50-51):1378-1382. 26720857 10.15585/mmwr.mm6450a3

[10] Department of Information Evidence and Research WHO Methods and Data Sources for Global Burden of Disease Estimates 2000-2015. Geneva, Switzerland: World Health Organization; 2017.

[11] Institute for Health Metrics and Evaluation GBD Profile: United States. Seattle, WA: Institute for Health Metrics and Evaluation; 2010.

[12] Murray CJ, Atkinson C, Bhalla K, ; US Burden of Disease Collaborators The state of US health, 1990-2010: burden of diseases, injuries, and risk factors. JAMA. 2013;310(6):591-608. 23842577 10.1001/jama.2013.13805 PMC5436627

[13] Substance Abuse and Mental Health Services Administration Opioid Use in the Older Adult Population. Rockville, MD: Substance Abuse and Mental Health Services Administration; 2017.

[14] Centers for Disease Control and Prevention Death investigation systems. https://www.cdc.gov/phlp/publications/coroner/death.html. Updated October 26, 2016. Accessed March 3, 2018.

